# Changes in audio-spatial working memory abilities during childhood: The role of spatial and phonological development

**DOI:** 10.1371/journal.pone.0260700

**Published:** 2021-12-14

**Authors:** Walter Setti, Luigi F. Cuturi, Giulio Sandini, Monica Gori

**Affiliations:** 1 Robotics, Brain and Cognitive Science (RBCS) Unit, Istituto Italiano di Tecnologia, Genoa, Italy; 2 Unit for Visually Impaired People (U-VIP), Istituto Italiano di Tecnologia, Genoa, Italy; Birkbeck College, UNITED KINGDOM

## Abstract

Working memory is a cognitive system devoted to storage and retrieval processing of information. Numerous studies on the development of working memory have investigated the processing of visuo-spatial and verbal non-spatialized information; however, little is known regarding the refinement of acoustic spatial and memory abilities across development. Here, we hypothesize that audio-spatial memory skills improve over development, due to strengthening spatial and cognitive skills such as semantic elaboration. We asked children aged 6 to 11 years old (n = 55) to pair spatialized animal calls with the corresponding animal spoken name. Spatialized sounds were emitted from an audio-haptic device, haptically explored by children with the dominant hand’s index finger. Children younger than 8 anchored their exploration strategy on previously discovered sounds instead of holding this information in working memory and performed worse than older peers when asked to pair the spoken word with the corresponding animal call. In line with our hypothesis, these findings demonstrate that age-related improvements in spatial exploration and verbal coding memorization strategies affect how children learn and memorize items belonging to a complex acoustic spatial layout. Similar to vision, audio-spatial memory abilities strongly depend on cognitive development in early years of life.

## 1. Introduction

In everyday life, we need to encode and temporarily store features of objects in our surrounding environment. When we need to recall an object’s position, spatial and semantic binding to the surroundings aid this task [1]. Such processes are accomplished through working memory (WM), a cognitive system involved in the maintenance and sequential manipulation of incoming, task-relevant information. High-level cognitive processes such as reasoning, learning, problem-solving, language comprehension, and motor planning also rely on WM [2,3]. Baddeley and Hitch first proposed the WM construct (i.e., the Multi-Component model) [3,4]; according to this pioneering hypothesis, WM consists of a tripartite structure with a central executive component and two domain-specific subsystems: the visuo-spatial sketchpad and the phonological loop. The central executive component is responsible for a wide range of regulatory functions, such as attentional control and reasoning [5–7]. The visuo-spatial sketchpad is specialized for maintenance of visual and spatial information [8–10]. The phonological loop is dedicated to storage of verbal information, and is composed of two subsystems: the phonological storage to hold verbal information and the articulatory loop to refresh verbal material through a process known as “rehearsal” [11]. Introduced after the tripartite system definition, the episodic buffer binds information across different components of working and long-term memory [12]. A more recent model considers these subsystems as distributed along a continuum rather than as discrete entities [13]. This continuum can be depicted as a cone-shaped model that develops along two dimensions: horizontal and vertical. The first refers to the type of information to be processed (e.g., verbal, visual, spatial); that is, the nature of the perceptual input. The vertical dimension indicates the level of control needed to accomplish a specific cognitive task, such as level of attention.

Previous research on the ontogenesis of WM has shown that the tripartite model proposed by Baddeley [4] is already in place by 4 years of age. The subsystems’ capacity increases until age 12 [5]. In other words, memory abilities continue to refine through infancy and childhood [14]. This observation supports a developmental pattern in which the prefrontal cortex, directly involved in WM processes, is one of the last cortical areas to reaches maturity [15–18]. Furthermore, WM abilities improve across childhood and adolescence. Performance in WM tasks reaches its peak during adulthood and begins to decline with advancing age [19]. Along this line, when it comes to memorization of spatialized information, both visual and acoustic, Vuontela and co-workers [17] observed a developmental trend in both sensory domains.

To date, development of visuo-spatial WM across age has been widely assessed through visually spatialized stimuli using visuo-spatial tasks such as the Corsi block-tapping task [20] and visual patterns tests [21]. Regarding audio-spatial memory, the *n-back* paradigm is the most used. In the context of the phonological contribution to WM, studies on children aged between age 6 and 11 years old showed developmental changes when required to encode visual items verbally. When processing information to be temporarily stored, such as a sequence of items to be remembered, young children are more bound to perceptual features of the stimuli and the encoding sensory modality. For example, in terms of visual features, infants and young children are likely to group objects based on visual features such as shape and colour rather than their semantic properties [5,14,22–25]. After age 8, children learn to use sub-vocal rehearsal to memorize visual items [26–28] and begin to rely on phonological codes to remember items. From this age onward, stored information is maintained mainly via the rehearsal process [23] and children develop the ability to combine visual information with phonological codes [24]. This ability is known as “semantic elaboration”, defined as the process of rehearsal of an item representation in words, which leads to better recall in many memory tasks [29–32]. However, semantic elaboration for non-verbal acoustic items has rarely been studied [33]; therefore, it remains unclear if the capacity to name a meaningful sound (i.e., animal call), may aid storing and recalling of auditory items.

The ability to remember spatial location relies on the formation of a mental representation of the spatial relationship with the item [33]. A fully developed spatial WM allows estimation of spatial positions by dividing the space into subspaces, based on physical barriers or functional grouping [34,35]. In other words, the actual position of an item is stored based on the boundaries of the enclosed space where the item resides. The processes used to encode locations take advantage of two types of spatial representations: metric and categorical [36–39]. Metric spatial representations specify distances considering a reference point (e.g., the house is 100 m left of the hospital). Categorical spatial representations refer to localization of objects within a larger region, without focusing on the exact coordinates (e.g., the house is at the centre of town); thus by building a complex representation of the scene that does not need the use of relative reference points, children begin to integrate metric and categorical representations after age 4 [40]. Depending on the specific ability, differential developmental trends can be observed until age 12, along with the ability to code spatial positions concerning distal landmarks [41–43].

While much research has focused on spatial exploration strategies and spatial WM skills in the visual domain, other sensory modalities can be used to encode the external world spatially. Spatial WM memory is, in essence, multisensory; thus, a compelling understanding of how this system processes, maintains and manipulates information needs to consider encoding sensory modalities other than vision. Despite the importance of short-term memory to successfully orient sound positions in space, most studies on auditory WM have focused on mnemonic processing of sound content [44–46] not location. Setti and colleagues [47] studied how exploring an auditory semantic scene could help recall spatialized sounds in healthy and blind participants. Their results highlight how visual experience is pivotal to gain semantic-related facilitation in processing acoustically-encoded spatial information. However, development-related changes in the strategies used to explore an acoustic spatial arrangement have been less investigated than those occurring in the context of visuo-spatial exploration.

To our best knowledge, only one study thus far has investigated the development of audio-spatial and visuo-spatial WM in typical children aged between 6 and 13 years of age [17]. Using a visual and acoustic version of the *n-back* test, where participants had to recall the position of previously presented spatial items, the authors observed that recall accuracy increased with age. Based on these results, the authors suggest that age-related improvements may be associated with recoding visual and auditory items into a verbal form, an ability that reaches maturity after age 8. Comparison of performance across modalities revealed that children performed visual tasks faster and more accurately than corresponding auditory tasks, leading authors to conclude that visual WM reaches functional maturity earlier than audio WM.

Up to now, such WM functions have been investigated mostly in the context of visuo-spatial processes. However, exploration of the surrounding space takes advantage of a multisensory environment; thus, is not only visual but also auditory. In particular, when visual information is degraded or unavailable (e.g. in a foggy environment or in the case of clinical blindness), auditory information is fundamental to orient and encode the surroundings. In previous studies comparing visual and auditory spatial WM [17], authors focused on the passive reception of stimuli. Conversely, the introduction of spatial exploration in an audio-spatial memory task not only has ecological value but may provide insights on potential memorization aids provided by semantic association [47]. Along these lines, we introduced the spatial exploration of an array of acoustic items to study the influence of development in the storage and pairing of spatialized sounds and their interaction with semantic associations, thus providing insightful understanding of exploratory and phonological strategies that are not investigated via the classical *n-back* test. Concerning the apparatus usually employed to evaluate cognitive abilities, in the audio-memory test we used a complex 2D spatial layout, which allowed us to consider the influence of stimuli spatiality on memory skills and, at the same time, gave us the ability to employ mental imagery strategies to memorize sound locations. Due to the lack of proper technologies, the influence of complex spatial arrangements on memory skills has not been fully investigated, especially in the early years of life. To test the ability to recall spatially displaced sounds in one condition (“call-call”) children were asked to pair identical animal calls (e.g., two barks). In the other experimental condition (“call-name”), we focused on the role of phonological codes in the recollection and semantic association of the acoustic stimuli. We asked participants to match the animal call with the name of the corresponding animal (e.g., a bark with a recorded voice saying “dog”). Our core assumption was that the development of spatial skills and memorization strategies occurring in the visual domain should also be reflected in audition. In detail, since the ability to integrate spatial metrics takes time to fully develop, we hypothesize that younger participants would suffer more limitations in building a coherent representation of sound locations. Furthermore, due to the spatial displacement of the sounds and the incomplete maturation of cognitive skills such as phonological codes and semantic elaboration, performance in the call-name condition should be lower in young children compared to older peers and the call-call condition. We expect to observe a developmental shift around the age of 8, also for audio-spatial memory skills.

## 2. Results

**Fig 1:**
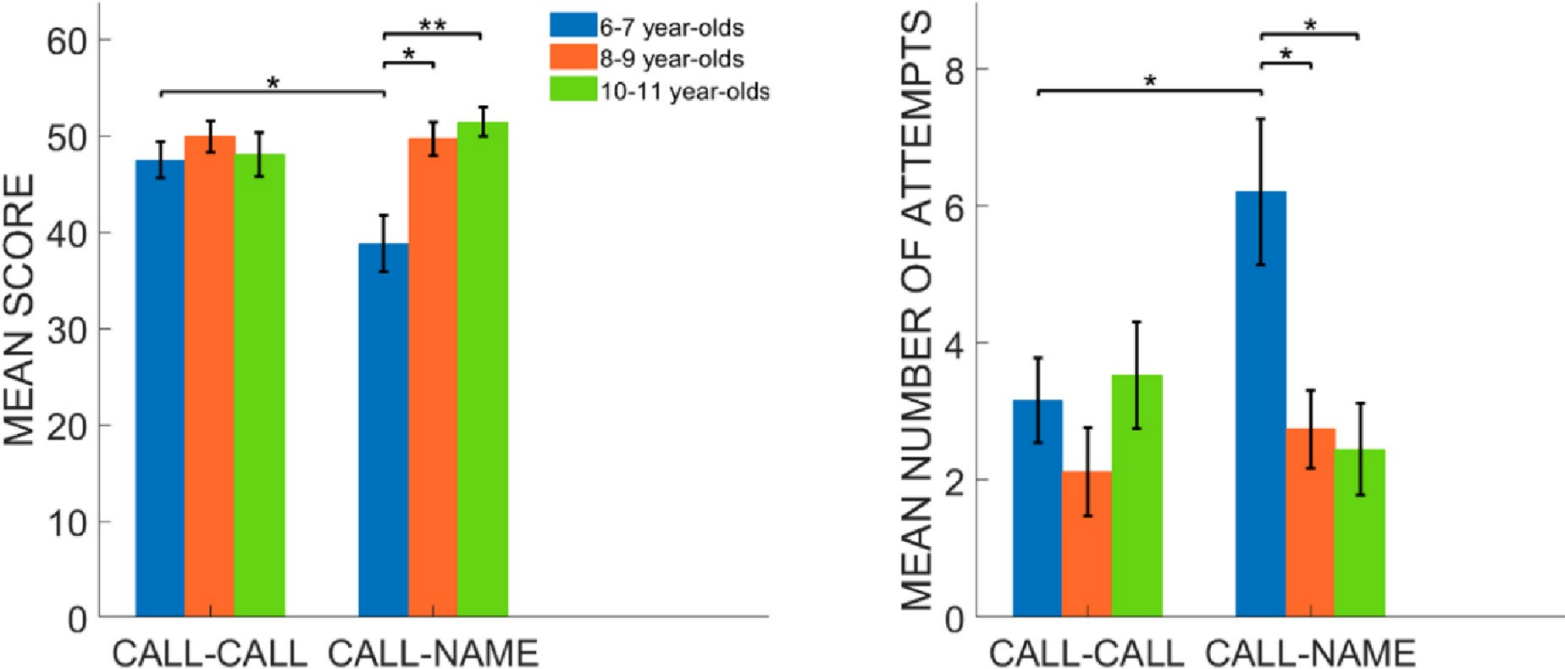
Performance analyses. Data are presented as mean across participants; error bars represent standard error. The left panel shows the results for the *score* parameter; the right panel represents the results for the *number of attempts* parameter. In the call-name condition, 6–7 year-olds reached a lower *score* and employed a greater *number of attempts* compared to the other two groups and the call-call condition. Asterisks indicate significant differences (see Results section for details). One asterisk (*) represents p < 0.5 Two asterisks (**) represent p < 0.01 and three asterisks (***) p < 0.001.

First, we ran outlier analyses for each investigated parameter; we excluded participants that reported outliers for at least one of the parameters (11 were excluded). Participants were re-arranged as follows: 6–7 years old, n = 18 (9 females, mean age: 6.39, std: 0.5), 8–9 years old, n = 12 (4 females; mean age: 8.54, std: 0.52), 10–11 years old, n = 14 (9 females, mean age: 10.2, std: 0.41). Data referring to the score followed a normal distribution. 6–7 year olds reached a lower score in the call-name condition than the other two groups and the call-call condition (Fig 1; left). The results showed a significant main effect of the *Group* (F (2, 41) = 5.51, p < 0.01, η² = 0.21), no significant effect given by the *Condition* (F (2, 41) = 2.38, p = 0.13), and a significant interaction Group*Condition (F (2, 41) = 5.26, p < 0.01, η² = 0.20). Given the significant interaction Group*Condition, we carried out follow-up ANOVA tests for the two factors separately. Regarding *Condition*, we performed a one-way ANOVA for the two conditions separately by comparing the three groups in the analyses. The results showed no significant difference between the groups in the call-call condition (F (2, 41) = 0.39, p = 0.67) but a significant difference in the call-name condition (F (2, 41) = 8.81, p < 0.001, η² = 0.3). As confirmed by results of post-hoc analyses, the youngest participants reached a lower score in the call-name condition compared to the age 8–9 group (unpaired t-test: t = 3.16, p = 0.012, df = 25.87, Cohen’s d = 1.1 *Bonferroni* corrected) and 10–11 year olds (unpaired t-test: t = 3.39, p < 0.01, df = 23.15, Cohen’s d = 1.29, *Bonferroni* corrected) and to the call-call condition (paired t-test: t = 2.76, p = 0.013, df = 17, Cohen’s d = 0.65). Regarding number of attempts, the data did not follow a normal distribution. Therefore, we carried out an analysis of variance based on permutations (*aovp()* R function). The results showed that 6–7 year olds needed more attempts to pair an animal call with the corresponding name after having discovered their positions on *ARENA* (see Fig 1 right panel) compared to the older peers. We observed a significant main effect of the *Group* (iterations = 5000, p = 0.017, η² = 0.1), no significant effect of the *Condition* (iterations = 51, p = 0.92) and a significant interaction between the two factors (iterations = 3608, p = 0.03, η² = 0.083). Given the significant interaction between the factors, we ran separate follow-up ANOVAs for each experimental condition by taking age group as between factor. In the call-call condition, we did not observe a significant effect of age group in the number of attempts to pair two discovered stimuli (iterations = 166, p = 0.39) while the call-name condition, ANOVA reported a significant influence of age (iterations = 5000, p < 0.01, η² = 0.23). Post-hoc analyses were carried out with Student’s t-tests based on permutations. As shown in Fig 1, in the call-name condition 6–7 year olds needed more attempts compared to the age 8–9 group (unpaired t-test based on permutations: Welsh t-statistic = 2.8649, p = 0.014, Cohen’s f^2^ = 0.99, *Bonferroni* corrected) and the 10–11 group (unpaired t-test based on permutations: Welsh t-statistic = 2.9838, p = 0.018, Cohen’s f^2^ = 1.03, *Bonferroni* corrected). Furthermore, only 6–7 year olds employed more attempts to end the call-name task than the call-call condition (paired t-test based on permutations: Welsh t-statistic = 2.4689, p = 0.016, Cohen’s f^2^ = 0.57 *Bonferroni* corrected).

**Fig 2:**
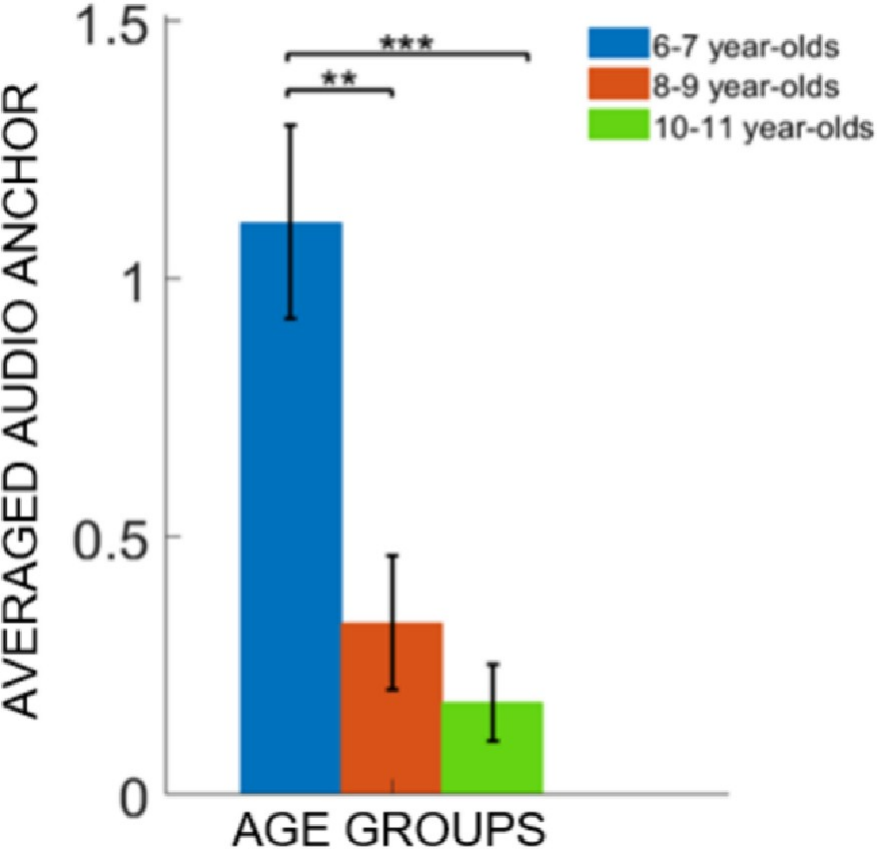
Audio-anchor. Data are presented as the mean across participants per each group; error bars represent the standard error. Regardless of the condition, 6–7 year-olds used the *Audio-anchor* exploration strategy more often compared to the other age groups. Significant comparisons between groups are represented. Two asterisks (**) represent p < 0.01 and three asterisks (***) p < 0.001.

Concerning the use of the audio-anchor (Fig 2), we found that regardless of the tested condition, 6–7 year olds started consecutive attempts by touching the same speaker more often than the other two groups. These analyses showed a significant main effect of the *Group* (iterations = 5000, p < 0.001, η² = 0.23) and no significant effect of *Condition* (iterations = 322, p = 0.25) or interaction between the two factors (iterations = 263, p = 0.41) was found. Post-hoc analyses revealed that, regardless of the experimental condition, 6–7 year olds relied more on the audio-anchor strategy to explore the device compared to the 8–9 (unpaired t-test based on permutations: Welsh t-statistic = 3.4281, p < 0.01, Cohen’s d = 0.86, *Bonferroni* corrected) and 10–11 year-olds (unpaired t-test based on permutations: Welsh t-statistic = 4.6083, p < 0.001, Cohen’s d = 1.1 *Bonferroni* corrected). These results seem to confirm the existence of a developmental cut off around age 8.

**Fig 3:**
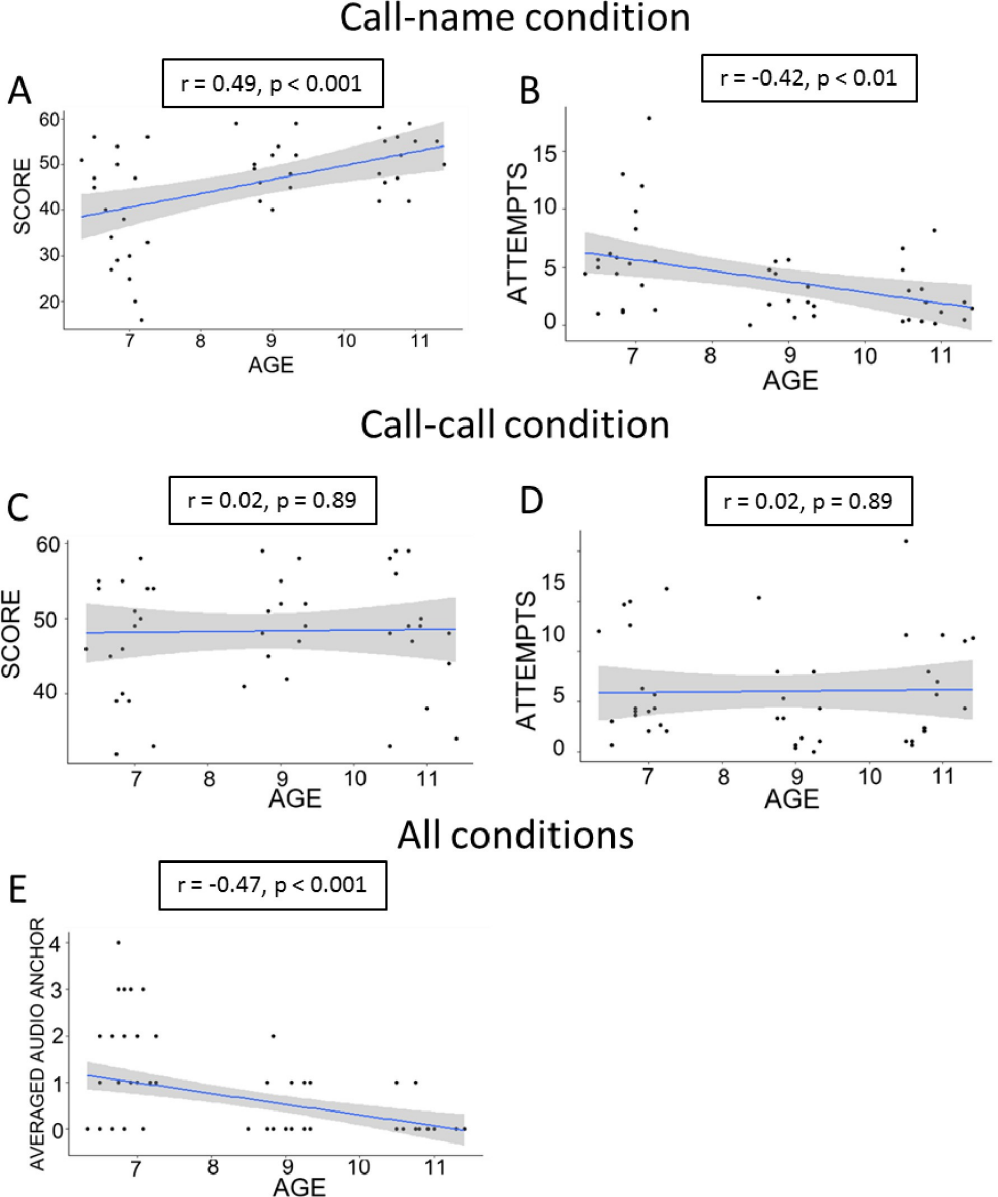
Linear regression analysis. Linear regression plot with 95% confidence intervals (shaded areas). The box on top of each plot shows the correlation and the significance level. Top row shows the linear regression in the call-name condition for the *score* (A) and the *number of attempts* (B). Middle row shows the linear regression in the call-call condition for the *score* (C) and the *number of attempts* (D). Bottom row shows the linear regression for both conditions for the *Audio-anchor* (E).

In order to better investigate this apparent trend in development, we performed a correlation analysis between age and three parameters (score, number of attempts and audio-anchor) wherein we treated age as a continuous variable by using decimal values. Specifically, we adopted a regression analysis with the continuous variable *Age* as a predictor. The ANCOVA score did not reveal any significant effect given by the *Condition* (F (1, 84) = 1.88, p = 0.17) but a significant main effect of *Age* (F (1, 84) = 8.66, p < 0.01, η² = 0.093) and a significant interaction Age*Condition (F (1, 84) = 7.627, p < 0.01, η² = 0.083). Given the significant interaction between these factors, we ran linear regression analyses separately for the two experimental conditions by considering the *age* as a predictor. We found that the value of the score reached by the children increased with *Age* in the call-name (r = 0.49, p < 0.001, Cohen’s f^2^ = 0.32) but not in the call-call condition (r = 0.022, p = 0.89) (Fig 3A and 3C). In other words, we found a significant positive correlation between age and score only in the call-name condition.

We did not find a significant effect given by the *Condition* (iterations = 1255, p = 0.08), but a significant main effect of the *Age* (iterations = 4483, p = 0.022, η² = 0.062) and a significant interaction Age*Condition (iterations = 3833, p = 0.027, η² = 0.071). Due to these significant interactions, we ran linear regression analyses separately for the two experimental conditions. Correlation analysis showed that the number of attempts employed to end the task decreased with age in the call-name condition (r = -0.42, p < 0.01, Cohen’s f^2^ = 0.22) but not the call-call condition (r = 0.022, p = 0.89) (Fig 3B and 3D; right). Finally, we evaluated the tendency to use the audio-anchor as an exploration strategy. Since these data did not follow a normal distribution, we ran ANCOVA based on permutations. The results highlighted a main effect of the *Age* (iterations = 5000, p < 0.001), no significant effect of *Condition* (iterations = 1846, p = 0.06) and no significant interaction Age*Condition (iterations = 51, p = 0.7). Post-hoc analysis was carried out by comparing the age groups in overall performance regardless of the tested experimental condition. The results are shown in Fig 3E. We found a negative correlation between age and tendency to use the audio-anchor (r = -0.47, p < 0.001, Cohen’s f^2^ = 0.35). Therefore, younger children tended to rely more on this exploration strategy by referring to all the locations as a fixed position in space.

## 3. Discussion

We investigated the development of audio-spatial exploration and memory abilities in pre-adolescent children. We focused on how mutual spatial relations and semantic binding among spatialized meaningful sounds in a memory task would change during childhood. Since visuo-spatial skills in the context of WM processes are known to reach maturity relatively late in development (i.e., pre-adolescence), we expected a similar pattern for audio-spatial WM, a cognitive component that has received substantially less attention compared to visuo-spatial WM. Our results show that the youngest group of participants (6–7 years old) tended to start consecutive attempts by touching the same speakers, thus anchoring their spatial exploration to previously explored items (i.e., the audio-anchor strategy; Fig 2). Such developmental trend seems to reflect the maturation of exploration strategies in using metric and categorical representations of space, a phenomenon known in the literature for visual layouts [38,40,48]. In this study, we developed a novel ad hoc task, and therefore could not compare our results with an exact equivalent in the visual domain. Thus, we mainly parallel audio-spatial development with the general development of spatial WM in the visual domain observed in the literature. The inclusion of semantic and spatial exploration strategies provides a more complex scenario than that drawn from pure WM tasks that provide a direct comparison of visual and auditory domain (e.g., the *n-back* test, see Introduction).

Furthermore, 6–7 year olds showed poorer audio-spatial memory abilities than older peers when items of different acoustic nature (i.e. spoken word and animal call or vice versa in the call-name condition) had to be matched. Such development-related differences reflect the maturation of the ability to exploit semantic association to pair spatialized semantically-related sounds with different acoustic features. By focusing on the development of spatial exploration strategies, we observed that, regardless of the experimental condition, 6–7 year olds start consecutive attempts by touching the same stimulus location more often than older participants. Thus, before age 8, children do not seem to account for mutual spatial relations between sound items to accomplish the task, but rather follow a sequential exploration pattern anchored to a reference point. Alternatively, older children may use a higher explorative strategy by more randomly choosing the item to start with compared to younger children, though such a strategy would have increased the overall number of attempts, which instead is lower in older children. Parallel to the visual processing of space, integration of nearby auditory spatial items might take time to fully develop. Specifically, in the visual exploration of spatial layouts, children at 4 years of age begin integrating two types of spatial representations [49]: metric, that is, fine-grained spatial information considering distance and direction from a reference point, and categorical, that is, a coarser representation of space taking into account a larger region of interest [36]. Depending on the specific spatial ability of interest, the use of either of these representations (or their integration) continues to develop until age 12 [41–43]. For instance, around 7 years old, children integrate space processing along two dimensions, while beforehand their metric representation is mostly anchored to a single dimension of space [48]. In our study, children younger than 8 likely took advantage of metric spatial representation for coding audio-spatial items. That is, they were more prone to explore the spatial layout by using a known reference. Such fine-grained space exploration, though improving precision while exploring the items, might indicate a lower ability to group and categorize space. In other words, after age 8, children might be better able to differentiate the auditory structure of items in spatial categories, thus improving the quality of their spatial exploration strategy with lower reliance on the anchoring to a known reference.

However, our investigation of the anchoring strategy has some limitations. An alternative interpretation of our results may take into account spatial uncertainty associated with the item selection. In other words, younger children may have been less certain about which item location among neighbouring slots contained the target item to be matched. In this sense, the anchoring strategy would be influenced by a low-order spatial encoding ability that in turn reflects on the spatial exploration strategy adopted to accomplish the task. To overcome this limitation, we employed methodological solutions to provide participants with highly discriminable items’ location (see grid description in 6.2 MATERIAL) whose presence was removed from the matrix once a match of items was discovered, thus leading to a progressive reduction of spatial uncertainty as the task proceeded. In this sense, we are confident the spatial exploration strategy is the best candidate explanation for the presence of development-related differences in the use of the audio-anchor. Nonetheless, future studies may test whether development related differences in spatial localization ability influence the exploration strategy adopted in an audio-spatial task by for instance leaving all items available during the task and specifically map the performed exploration route.

The observed developmental shift at around 8 years old in spatial exploration abilities fits well with the cross-sensory calibration theory in the context of auditory spatial representation [50,51]. While 4-year-old children can integrate visually encoded metric and categorical spatial representation [49], our results indicate that categorical representation in the processing audio-spatial seems to emerge after age 8. In line with the cross-sensory calibration theory, visual experience might be needed to build a proper audio spatial representation [52–54]. The absence of visual-auditory integration in spatial processing may indeed underlie the spatial exploration strategy employed by younger children. As age and visual experience increase, cross-sensory calibration of visual on auditory processing of space may lead to more functional spatial exploration at a lower cognitive cost, as indicated by reduced use of a reference to solve our memory task in older children. In the context of spatial representations, the employment of a bisection task testing the development of complex spatial representations showed meaningful changes after the age of 12 years old, mostly referring to auditory and visual attraction to either temporal and spatial domains, respectively [52,53]. While the bisection task requires participants to build a complex spatial representation, our task shows that when no requirements are provided, the use of spatial relationships between items spontaneously occurs more likely in children older than 8. Thus, our findings suggest that spatial exploration in the context of auditory-only processing improves already at 8 years old, with spatial exploration a simpler process that may provide the basis for further developing more complex elaborations of space. Considering the integration of visual and auditory information, the absence of visual input in our study allowed us to isolate the processing of auditory-only spatial items and test development-related differences within this sensory modality. However, depending on the multisensory integration level, the combination of visual and auditory information may improve the ability to explore and remember the position of items. In this sense, further studies with visual-auditory stimuli could be pursued to investigate the role of cross-sensory calibration in spatial exploration deeply.

Our findings support the hypothesis that audio-spatial WM abilities take more time to develop compared to vision-based processes. Although we did not test a visual version of our task, previous literature has demonstrated slower developmental trends for the auditory domain compared to vision by comparing performance to a visual and an auditory version of the *n-back* test [17]. The abovementioned developmental shift in exploration strategies observed at 8 rather than 4 years of age supports this hypothesis. Nonetheless, the substantial methodological differences between the audio-memory and the *n-back* (e.g. the number of spatialized items; 12 vs. 3 and the inclusion of semantic and spatial exploration strategies in the audio-memory) require caution in drawing conclusions from the comparison between the two tasks. In this sense, a visual version of the audio-memory would be desirable to provide a better understanding of sensory domain dependent developmental trends that we may predict would follow different velocities depending on the investigated sensory domain.

In this study, we unveiled development-related differences in the use of semantic association to pursue memorization and pairing of auditory spatialized items. Semantic associations were tested by taking advantage of the relation animal-call (e.g., dog barks) clearly understood by all children tested in our study, as expected based on pilot studies and previous literature that reported the presence of general ability to recall names based on associative properties of the items to be recalled, though in the visual rather than auditory modality [55,56]. Nonetheless, we found clear developmental trends in our auditory spatial memory paradigm when the semantic relationship between audio-spatial items could aid memorization. Specifically, younger children needed more attempts to pair the sounds and reached a lower score in the call-name condition compared to older peers. This difference is present between conditions, as 6–7 years old performed poorer in the call-name compared to the call-call condition (Fig 1). Therefore, it seems that the rapidity of association of two unidentical sounds is slower in the youngest group of children. Differential maturation of the use of the phonological loop might underlie such observed differences. The process of “naming”, that is, the assignment of a name to an item involves a semantic elaboration that may facilitate spatial memory and the formation of cognitive maps [33]. While in the call-call condition, the sounds to be matched had the same semantic and acoustic features, in the call-name condition animal calls and names were by definition acoustically different. Previous research on word memorization has pointed towards a developmental shift from phonological to semantic processing of words around 8 years of age [57]. When asked to memorize a set of words, children younger than 8 tend to focus more on acoustic features such as rhyming related words, rather than the semantic meaning of the items (the concept they refer to). Along these lines, in our study, 9–10 year olds likely made greater use of semantic associations compared to younger children, resulting in facilitation in accomplishing the matching task, and thus better recalling the spatial location of the auditory items. After age 8, children also gain the ability to memorize objects by assigning labels and subvocally rehearse a given label to the object [58,59]. For instance, memorization of an object such as a bottle can be accomplished by naming the object as “bottle” instead of focusing on its physical properties such as its cylindrical shape or its colour. Supporting this view, previous studies showed that 6–7 year-olds mostly rely on the visuo-spatial sketchpad to recall visual stimuli, focusing on pictorial features such as shape and orientation [23]. Older children are more likely to recall visual inputs into a phonological form by employing the rehearsal process as this ability is consolidated around 8 years of age [23]. It is likely that this group of children in our study took advantage of the subvocal rehearsal to prevent the decay of the stimuli to be remembered [60]. In other words, the concept describing both the animal name and the call was used as a link between the items to successfully recall their position. In contrast, younger children (6–7 years old) are overall more likely to be anchored to the perceptual, acoustic features of the sounds. In this sense, they did not take advantage of the underlying semantic concept binding the items to be matched such that their performance was affected by the different acoustic properties of the items in the call-name condition. Supporting this view, we observed comparable performance for children of all ages in the call-call condition, where pairing could be based mostly on the acoustic properties of the sound regardless of the underlying semantic meaning. Alternatively, it can be argued that auditory spatial memory for a task as simple as call-call matching is no longer substantially developing after age 6–7 years. The developmental consistency observed in the performance to this task suggests that children’s’ ability to match identical calls may simply not be affected by the abovementioned reduced ability to use semantic association and subvocal rehearsal to accomplish the task. In this sense, the simple ability to match unidentical items may be influential in the matching process regardless of the spatialization of items. Here, we aimed to prevent such possibility by choosing items that are easily recognizable by children of all ages (see pilot studies in 6.2 MATERIALS); however, future studies may further investigate this aspect, for instance by testing whether children’s’ capability to associate sounds predicts their ability in matching spatialized items.

Altogether, our findings suggest that the age of 8 years old may represent an approximate switching point in the ability to use semantic information to bind items, not only for general memorization purposes [57] but also when these are spatially displaced [61]. The outcome of such a developing system may be reflected by age-related improvements in the ability to explore the surrounding space [62,63] and take advantage of landmarks and environmental properties [61] to build a functional representation of space.

Although our results suggest that spontaneous use of the subvocal rehearsal is likely to take place in children older than 8 years old when performing an audio-spatial WM task, we did not specifically compare performance with a task blocking participants from using such a strategy [64,65]. Further research could overcome the limits of our study, for instance by evaluating the use of naming acoustic objects, testing how interference with the subvocal rehearsal process (e.g. by repeating the word “the” while performing the task) differentially affects children’ performance depending on their age. Similarly, it can be studied whether this phenomenon extends to different categories of objects or across sensory modalities.

## 4. Conclusions

Typical development of audio-spatial memory abilities is influenced by the maturation of exploration strategies and cognitive abilities such as the use of semantic binding between items. Before age 8, developing spatial skills prevent young children from employing functional spatial exploration strategies to encode an auditory-only array. Exploratory behaviour seems to be linked to the use of reference points, such as previously explored location, suggesting that young children rely on a more sequential and relative representation of the acoustic environment rather than taking advantage of a more absolute and coarser representation of the explored space. Additionally, semantic information aids memorization to a greater degree in the older children compared to younger peers, suggesting that spatial exploration and likely phonological loop maturation may be interlaced, being both fundamental to accomplish audio-spatial WM tasks. In this context, the presence of a probable developmental shift at 8 years of age, posits this age as a critical point in the maturation of spatial WM processes.

In this study, we designed tasks in the form of a game especially suitable for children. This procedure has the potential to inspire future protocols aimed at strengthening descriptive and semantic associations between concepts and their meanings, especially in sensory and cognitive disabilities. Audio-spatial memory skills can be trained in case of early visual deprivation, providing a new way of teaching and learning semantic and spatial associations through the auditory channel. Finally, our findings can act as a launchpad for studying brain mechanisms underlying cognitive and spatial abilities development through the auditory channel, by involving a higher number of controls.

## 5. Material and methods

### 5.1 Participants

55 children recruited from a local school based in Genoa (Italy) took part in the experiments and were divided into three age groups: 6–7 years old (n = 23, 11 females, mean age: 6.87, std: 0.27), 8–9 years old (n = 15, 6 females; mean age: 8.96, std: 0.26), 10–11 years old (n = 17, 11 females, mean age: 10.83, std: 0.32). After the outlier analysis we removed 5 children, Participants did not have any cognitive or sensory impairments. The ethics committee of the local health service (Comitato Etico, ASL 3, Genoa, Italy) approved this study. We conducted all experiments in accordance with the Declaration of Helsinki. All children agreed to take part in the experimental session, and we obtained parental informed written consent in all cases. Before entering the testing room, participants were informed they could interrupt the experiment at any time; however, no one asked for a pause.

### 5.2 Material

**Fig 4:**
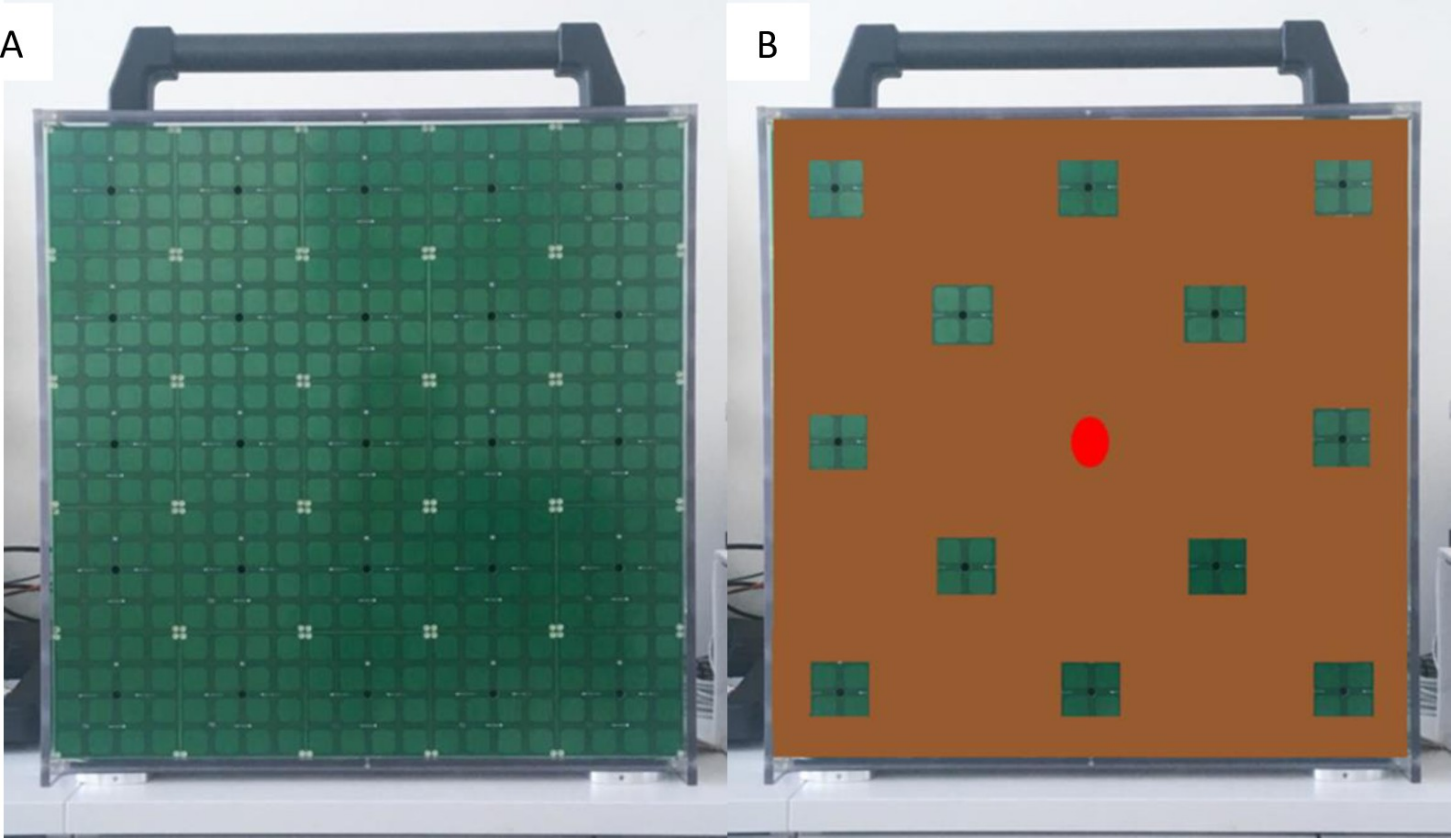
ARENA. **A)** The device measures 50 cm x 50 cm and consists of 25 speakers embedded in blocks covered by tactile sensors. Tactile sensors cover the blocks. **B)** The cardboard grid placed over the ARENA is represented. The grid is composed of 12 free slots. The feedback sounds were emitted from the speaker indicated by the red dot.

The task was carried out by means of an audio-tactile device (50 cm x 50 cm) called *ARENA* [66]. This device consists of 25 haptic blocks, each made of one loudspeaker covered by tactile sensors (16 per block, 2 cm x 2 cm each; Fig 4A). We placed a cardboard grid on the surface of the device (Fig 4B), leaving 12 uncovered squares measuring 4 x 4 cm and a small aperture (0.5 x 0.5 cm) in the centre to allow audibility of the central speaker. The grid apertures were smaller compared to the underlying haptic blocks to help participants in distinguishing stimuli locations (Fig 4B). The device was vertically oriented and positioned in front of the participant. Based on pilot studies, we selected 6 target animal calls that children can easily identify (See S1 Table in Supplementary Material for details regarding sound stimuli). For animal calls, we used audio mp3 files downloaded from a royalty-free website (https://freesound.org/). Each animal call lasted 2.5 seconds. In the pilot studies, we presented sighted volunteers with a set of spoken words (i.e., the six target animal calls of S1 Table plus other four sounds presented in S5 Table in the Supplementary Material section). We chose the six sounds that the participants more easily recognized. The spoken word duration was shorter and depended on the specific word (see S1 Table in Supplementary Material for details regarding duration of spoken words). As we conducted the experiment in Italy, the words in the call-name condition were pronounced in Italian language.

All subjects sat at a distance of ~20 centimetres from *ARENA*. To exclude the influence of vision and of the setup/room observation on the performance [67], all subjects were blindfolded with blackened swim goggles positioned over a sleeping mask before entering the room and throughout the experimental session. None of the participants saw or interacted with the device before the test. The researcher adjusted the subjects’ position so that the centre of the device was at eye level for each participant. Through the experiment, children remained seated. Before the experiment, we made sure that all participants could reach the corners of the device.

### 5.3 Experimental procedure

**Fig 5:**
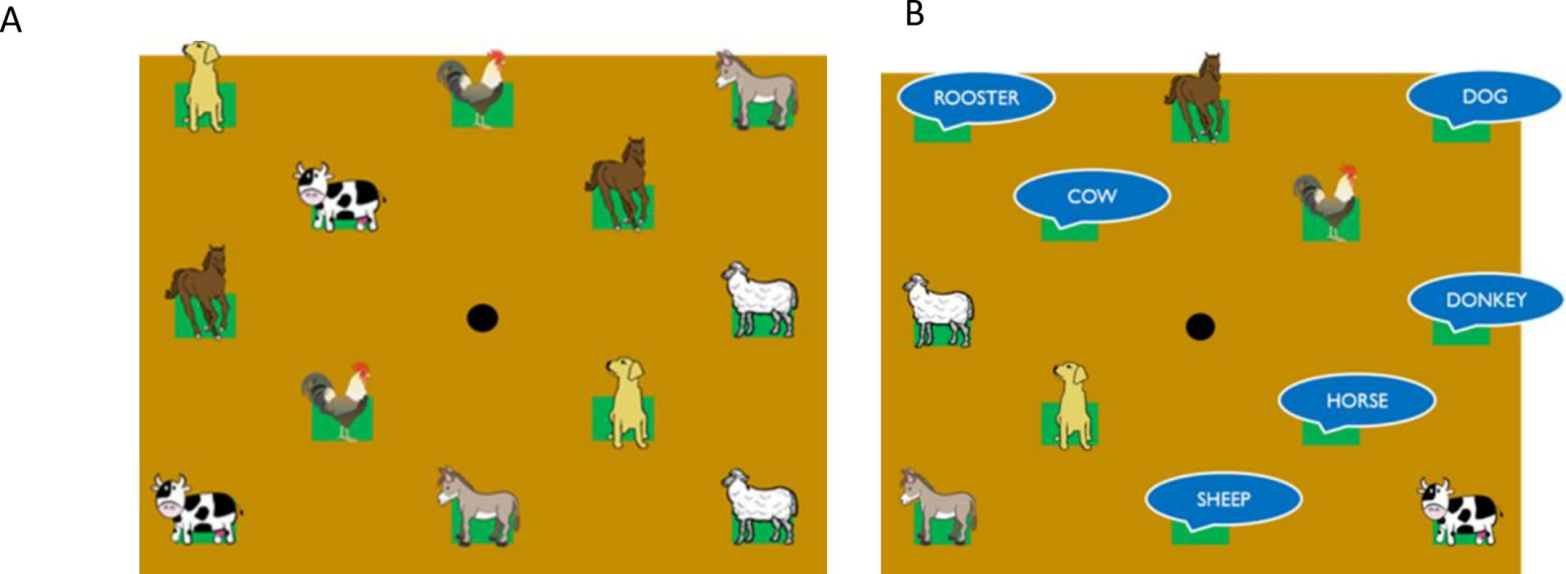
Experimental conditions. A) In the call-call condition, we asked the child to a couple animal calls. B) In the call-name condition, the animal call and the name of the animal had to be paired.

In designing the task, we took inspiration from “Memory”, a card game wherein the aim is to find pairs among an even number of cards. In the traditional version of the game, the cards have two sides: a visible side that identical for all cards, and a covered side showing images that are identical for pairs of cards. At the beginning of the game, the player turns all cards to the covered side. The player then flips two cards: if they match, the pair is removed from the group of cards on the table. Otherwise, they are turned back onto the covered side. The game ends when the player finds all matching pairs. In our study, instead of cards, we used sounds spatially displaced on a vertical plane. We designed our study with two experimental conditions based on the nature of the audio stimuli. In the call-call condition, we asked children to find pairs of animals’ calls (e.g., two identical dog barks). Instead, in the call-name condition, the participant had to pair the animal call with a recorded voice saying the animal’s name (e.g., the bark and the spoken word “dog”). We presented the two experimental conditions (Fig 5) in separate blocks, and counterbalanced the order of presentation across participants. In both experimental conditions, the study required children to find pairs of two sounds related to the same animal in a series of attempts until they discovered all pairings. Participants used the index finger of their dominant hand to touch two haptic blocks consecutively in each attempt. When a haptic block was touched, a sound was emitted; children were required to remember the location of the stimulus. The child had to wait for the sound to finish before touching another haptic block. Each time the child matched a pair, the experimenter placed a cardboard cover on the two apertures over the haptic blocks. Thus, the slots were hidden by the cardboard squares and the previous sounds were replaced by a recorded voice saying “NO”. Although the experimenter covered paired locations with cardboard patches, participants could accidentally touch two already paired locations. In these cases, we excluded the attempt from analysis. If two stimuli instead were matched, a “TADA” sound was emitted from the central speaker.

Before the test began, together with the child, the experimenter counted the open squares on the cardboard grid by guiding the participant’s dominant hand over it. Afterward, participants could freely explore the grid with both hands with no time restrictions until they felt confident with the use of the device. Confidence was verbally assessed before proceeding with the test. In this phase, we presented no sounds to the participant. After orientation, the experimenter explained the task by recalling the rules of the original memory game. Children were instructed to pay attention to the position of the sounds to identify the pairs of stimuli in the least number of attempts. After a successful attempt at encouraging children to perform in the best possible way, the feedback sound emitted. The researcher explained to the participants that the corresponding sound would be emitted when they touched a haptic block. Afterward, the child was presented with each animal call emitted from the central speaker and verbally identified the corresponding animal. As expected based on pilot studies (see 6.2 MATERIAL and Supplementary Material), all children successfully identified each call by naming the corresponding animal. In each experimental condition, before starting the test, the experimenter conducted a practice session with the child to familiarize them with the task. In this session, the experimenter guided each child’s dominant index finger starting from the central haptic block, first on two unpaired and then on two paired sound items that belonged to the 6 target sounds. The experimenter presented each participant with the feedback sounds for correct coupling and incorrect touch of a covered haptic block in the practice session. For the main experiment, we changed the position of the sounds used in the practice session to avoid potential learning effects. The test started at the end of this practice session. As in the original version of the memory game, participants could find any pair they liked with no imposed order by the experimenter. While the experiment was self-paced, it lasted approximately 20 minutes for each child. Before beginning of the test, the experimenter made sure the participant knew the card game “Memory” rules. Since the call-call condition (i.e., a pairing of identical animal calls) was more similar to the game, the task was easier to accomplish than the call-name condition, which increases in complexity. To avoid differences that might arise because of this complexity, we decided to let all children start with the call-call condition, which we expected to be more familiar to children and then increase complexity in the task with the call-name condition.

### 5.4 Data analysis

To quantify subjects’ performance according to the memory task, we focused on three parameters related to both memory skills and spatial exploration aspects:

1. **Score.** The *score* takes into account the frequency of touches on the same speakers: the more the participant returns on the same stimulus location, the lower the *score* (Fig 6). The final *score* is an index calculated over the global performance of the child in the test. We calculated the *score* as follows: in the first attempt, when a participant selected two stimuli positions for the first time, the *score* was calculated as 0. Since there is no influence of memory processes, first touches had no impact on the overall *score*. During subsequent attempts, if one or both stimuli positions were already touched in the previous attempts, the *score* decreased by 1 or 2, respectively. The tendency to return on these positions indicates that the participant failed to remember the sounds’ locations despite having already discovered them. When a participant found a pair, the *score* increased by 10, regardless of the number of touches on each speaker. Fig 6 illustrates an example of the *score*’s evaluation and the corresponding formula.

**Fig 6:**
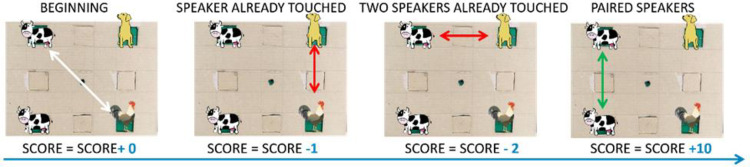
Example of score calculation. The score is an index that decreases when the child returns to the speakers previously touched. When the child touches two speakers for the first time, the score equals 0. If they have already touched one or both the speakers, the score decreases by 1 or 2, respectively. When a pair is found, the score increases by 10. In the example, if the starting value were equal to 0, the final score would have been: 0–1–2 + 10 = 7. Depicted animals were downloaded from a royalty-free images web archive (https://publicdomainvectors.org/).
2. **Number of attempts.** For each couple of sounds to be paired with the number of attempts, we quantified the number of attempts to pair with the two stimuli once positions were discovered. The higher the value, the more attempts the participant needed to pair two already-discovered stimuli. This parameter provides a measure of the ability to maintain the spatial location of the stimuli in memory in order to accomplish the task. We counted the number of attempts by starting when both sounds’ locations were discovered.
3. **Audio-anchor.** The *Audio-anchor* provides a measurement of the exploration strategy. It accounts for how many consecutive attempts the child begins by touching the same speaker (Fig 7). As this strategy is increasingly adopted, the index increases. For instance, suppose that the child encounters the dog bark and the donkey bray. If they began the following attempt again from the same dog’s sound position, the *Audio-anchor* index would increase by one. The index continues to increase until the child starts an attempt by touching a different stimulus position. The *Audio-anchor* provides a measurement of how well children build their spatial representation of sound disposition. The greater the index, the less is the use of the mutual spatial relationship between the sounds to accomplish the task.

**Fig 7:**
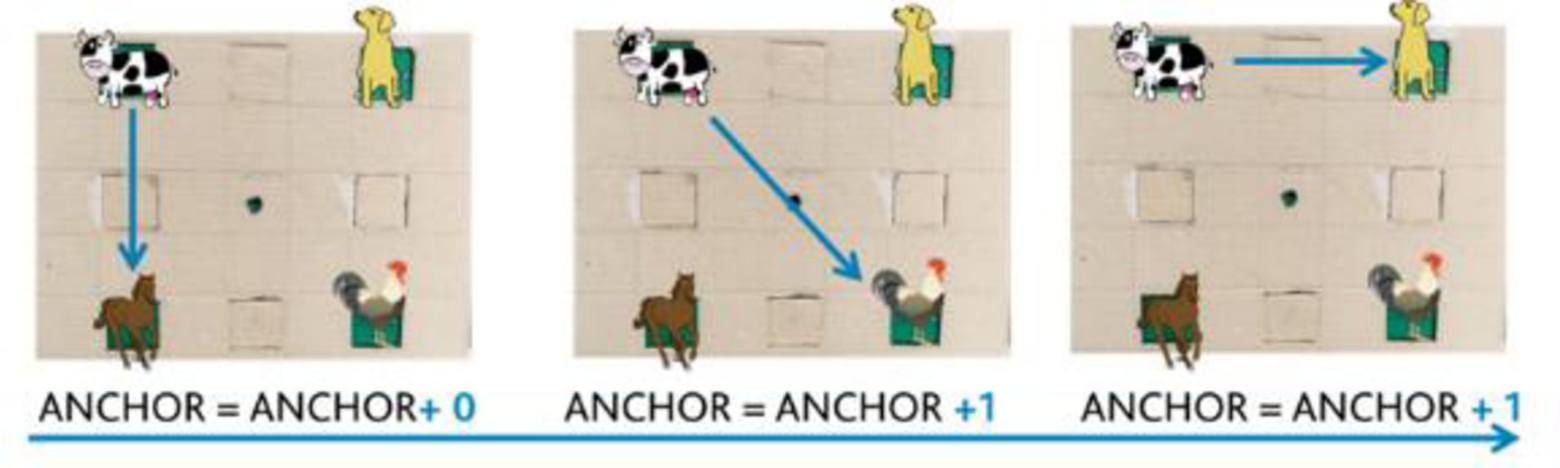
Example of Audio anchor calculation. The index equals 0 at the beginning of the test. The index increased correlated with more attempts started with the same speaker. In the represented example, the final value would have been: 0+1+1 = 2. Depicted animals were downloaded from a royalty-free images web archive (https://publicdomainvectors.org/).

### 5.5 Statistical analyses

Statistical analyses were carried out to investigate differences in the behavioural performance between the two experimental conditions. First, we performed an outlier analysis for each group of participants where we excluded those participants whose data deviated more than 2 standard deviations (SD) in at least one of the parameters. To test the hypothesis of a developmental trend in exploring the complex acoustic structure and in pairing the stimuli with different acoustic features (i.e., the call and the name), we binned the age of participants into three groups: 6–7, 8–9 and 10–11 year olds. Analyses were carried out in RStudio. To verify normality, we ran the Kolmogorov-Smirnov test. We then carried out 2-way (3 x 2) mixed measures analyses of variance (ANOVAs), with *Group* (either 6–7, 8–9 and 10–11 year olds) and *Condition* (either call-call or call-name) as between and within factors, respectively. In the case of significant interactions between the factors, we carried out follow-up ANOVAs. If the data did not follow a normal distribution, we used permutation tests to obtain p-values for linear models (*lmperm()* package) [68]. Two-tailed Student’s t-tests (paired and unpaired) were run in post-hoc analyses, and *Bonferroni* correction was used to correct for multiple comparisons (corrected p < 0.05 was considered significant) by multiplying the p-value by the number of tests performed.

To determine the relationship between age and memory skills and spatial exploration strategy, we ran analyses of the covariate (ANCOVAs) with *Age* expressed in decimal values as a covariate and *Condition* (either call-call or call-name) as within factor. Multiple linear regression analyses were carried out post-hoc to test the influence of age on the overall performance after verifying that the data fulfilled the necessary criteria for this type of analysis. The predicted variables were the score, the number of attempts and the audio-anchor. Age was considered as a predictor variable in all cases. In case of significance, effect sizes were calculated for each analysis. For ANOVAs and ANCOVAs, we calculated effect size in terms of partial eta-squared η² following the common interpretation for the effect size (small, η² > = 0.01; medium, η² > = 0.06; large, η² > = 0.14). For the t-tests, we calculated the Cohen’s d value and for linear regressions the Cohen’s f^2^ (for both parameters: small, d or f^2^ > = 0.2; medium, d or f^2^ > = 0.5; large, d or f^2^ > = 0.8).

## Supporting information

S1 Table Length of the spoken words used in the call-name condition. In the task, we used the terms indicated in the second column (in [removed for review purposes] language).
(DOCX)

S2 Table Effect of the gender on the score. The results of the ANCOVA do not highlight any significant main effect nor interaction of the gender with the Condition or the Age.
(DOCX)

S3 Table Effect of the gender on the number of attempts. The results of the ANCOVA do not highlight any significant main effect nor interaction of the gender with the Condition or the Age.
(DOCX)

S4 Table Effect of the gender on the audio-anchor. The results of the ANCOVA do not highlight any significant main effect nor interaction of Gender with Condition or Age.
(DOCX)

S5 Table Length of the spoken words used in the pilot studies. In the task, we used the terms indicated in the second column (in [removed for review purposes] language).
(DOCX)

S1 Dataset (7Z)
