# Supplementary material for: Changes in audio-spatial working memory abilities during childhood: The role of spatial and phonological development
Source: PLoS One. 2021 Dec 14;16(12):e0260700. doi: 10.1371/journal.pone.0260700 (PMC8670674; doi:10.1371/journal.pone.0260700)
Supplement: S2 Table — The results of the ANCOVA do not highlight any significant main effect nor interaction of the gender with the Condition or the Age. (DOCX) [file pone.0260700.s002.docx]

|  | DF | Sum Squares | Mean Square | F-Value | Pr(>F) |
| --- | --- | --- | --- | --- | --- |
| Age | 1 | 586 | 585.9 | 7.690 | 0.00691 ** |
| Condition | 1 | 114 | 113.6 | 1.492 | 0.22557 |
| Gender | 1 | 8 | 7.9 | 0.104 | 0.74757 |
| Age*Condition | 1 | 639 | 639.2 | 8.390 | 0.00486 ** |
| Age*Gender | 1 | 5 | 5.0 | 0.066 | 0.79773 |
| Condition*Gender | 1 | 6 | 6.2 | 0.081 | 0.77636 |
| Age*Condition*Gender | 1 | 0 | 0.4 | 0.05 | 0.94419 |
| Residuals | 80 | 6095 | 76.2 |  |  |

**Table S2:** Effect of the gender on the score. The results of the ANCOVA do not highlight any significant main effect nor interaction of the gender with the Condition or the Age.
