# Supplementary material for: Changes in audio-spatial working memory abilities during childhood: The role of spatial and phonological development
Source: PLoS One. 2021 Dec 14;16(12):e0260700. doi: 10.1371/journal.pone.0260700 (PMC8670674; doi:10.1371/journal.pone.0260700)
Supplement: S3 Table — The results of the ANCOVA do not highlight any significant main effect nor interaction of the gender with the Condition or the Age. (DOCX) [file pone.0260700.s003.docx]

|  | DF | Sum Squares | Mean Square | F-Value | Pr(>F) |
| --- | --- | --- | --- | --- | --- |
| Age | 1 | 51.5 | 51.46 | 6.94 | 0.01 * |
| Condition | 1 | 21.6 | 21.55 | 2.867 | 0.0925 |
| Gender | 1 | 1.7 | 1.74 | 0.23 | 0.63 |
| Age*Condition | 1 | 24.4 | 24.42 | 3.285 | 0.07 |
| Age*Gender | 1 | 2.3 | 2.27 | 0.306 | 0.59 |
| Condition*Gender | 1 | 0.5 | 0.52 | 0.07 | 0.79 |
| Age*Condition*Gender | 1 | 0.1 | 0.05 | 0.007 | 0.93 |
| Residuals | 80 | 594.5 | 7.43 |  |  |

**Table S3:** Effect of the gender on the number of attempts. The results of the ANCOVA do not highlight any significant main effect nor interaction of the gender with the Condition or the Age
