# Supplementary material for: Changes in audio-spatial working memory abilities during childhood: The role of spatial and phonological development
Source: PLoS One. 2021 Dec 14;16(12):e0260700. doi: 10.1371/journal.pone.0260700 (PMC8670674; doi:10.1371/journal.pone.0260700)
Supplement: S4 Table — The results of the ANCOVA do not highlight any significant main effect nor interaction of Gender with Condition or Age. (DOCX) [file pone.0260700.s004.docx]

|  | DF | Sum Squares | Mean Square | F-Value | Pr(>F) |
| --- | --- | --- | --- | --- | --- |
| Age | 1 | 13.87 | 13.86 | 20.698 | 1.89*10^-5 *** |
| Condition | 1 | 1.92 | 1.92 | 2.867 | 0.0943 |
| Gender | 1 | 0.04 | 0.04 | 0.06 | 0.80 |
| Age*Condition | 1 | 0.03 | 0.026 | 0.039 | 0.84 |
| Age*Gender | 1 | 0.05 | 0.054 | 0.08 | 0.778 |
| Condition*Gender | 1 | 3.26 | 3.26 | 4.863 | 0.06 |
| Age*Condition*Gender | 1 | 0.32 | 0.319 | 0.476 | 0.4922 |
| Residuals | 80 | 53.60 | 0.670 |  |  |

**Table S4:** Effect of the gender on the audio-anchor. The results of the ANCOVA do not highlight any significant main effect nor interaction of Gender with Condition or Age.
