# Supplementary material for: Changes in audio-spatial working memory abilities during childhood: The role of spatial and phonological development
Source: PLoS One. 2021 Dec 14;16(12):e0260700. doi: 10.1371/journal.pone.0260700 (PMC8670674; doi:10.1371/journal.pone.0260700)
Supplement: S5 Table — In the task, we used the terms indicated in the second column (in [removed for review purposes] language). (DOCX) [file pone.0260700.s005.docx]

| ENGLISH WORD | PRESENTED LANGUAGE [Removed for review purposes] | DURATION (SEC) |
| --- | --- | --- |
| DOG | [Removed for review purposes] | 0.65 |
| COW | [Removed for review purposes] | 0.8 |
| DONKEY | [Removed for review purposes] | 0.85 |
| HORSE | [Removed for review purposes] | 1 |
| SHEEP | [Removed for review purposes] | 0.7 |
| ROOSTER | [Removed for review purposes] | 0.9 |
| BIRD | [Removed for review purposes] | 0.56 |
| DEER | [Removed for review purposes] | 0.71 |
| HEN | [Removed for review purposes] | 0.62 |
| CRICKET | [Removed for review purposes] | 0.7 |

**Table S5:** Length of the spoken words used in the pilot studies. In the task, we used the terms indicated in the second column (in [removed for review purposes] language).
